# Tanshinone IIA attenuates Aβ-induced neurotoxicity by down-regulating COX-2 expression and PGE2 synthesis via inactivation of NF-κB pathway in SH-SY5Y cells

**DOI:** 10.1186/s40709-019-0102-1

**Published:** 2019-11-12

**Authors:** Lijiao Geng, Wei Liu, Yong Chen

**Affiliations:** Department of Neurology, Huaihe Hospital of Henan University, No. 357 Ximen Street, Kaifeng, 475000 China

**Keywords:** Tanshinone IIA, Amyloid-β, COX-2, PGE2, NF-κB pathway, Alzheimer’s disease

## Abstract

Amyloid-β (Aβ)-induced neurotoxicity is a major pathological mechanism of Alzheimer’s disease (AD). Tanshinone IIA (Tan IIA), extracted from traditional Chinese herb *Radix salvia miltiorrhiza*, possesses anti-oxidant and anti-inflammatory actions, as well as neuroprotective effects. The present study aims to explore the possible mechanism by which Tan IIA attenuated Aβ-induced neurotoxicity. Exposure of SH-SY5Y cells to different concentrations of Aβ led to neurotoxicity by reducing cell viability, inducing cell apoptosis and increasing neuroinflammation in a dose-dependent manner. Moreover, Aβ treatment promoted cyclooxygenase-2 (COX-2) expression and Prostaglandin E2 (PGE2) secretion, and activated nuclear transcription factor kappa (NF-κB) pathway in SH-SY5Y cells. However, pretreatment of SH-SY5Y cells with Tan IIA prior to Aβ prevented these Aβ-induced cellular events noticeably. These data suggested that Tan IIA exerted its neuroprotective action by alleviating Aβ-induced increase in COX-2 expression and PGE2 secretion via inactivation of NF-κB pathway.

## Background

Neurodegenerative diseases represent the progressive dysfunctions and neuron losses in the central nervous system, pathologically identified by the accumulation and misfolding of disease-specific proteins in particular part of the brain [1]. Alzheimer’s disease (AD), one of neurodegenerative disorders, is the most common form of dementia among the elderly, characterized clinically by behavioral, cognitive, and memory deficits [2]. The remarkable features in the brains of AD patients are extracellular deposition of amyloid-β (Aβ) plaques, intracellular neurofibrillary tangles and extensive neuronal loss in the brain. Several lines of evidence suggest that Aβ may be the critical factor in AD pathogenesis [3]. Multiple pathogenic processes, including oxidative stress, inflammatory responses, and apoptosis, have been revealed to be implicated in Aβ-induced neurotoxicity [4–6]. So far, it is generally accepted that Aβ can contribute to neurodegeneration and neuronal loss in AD. Therefore, the inhibition of Aβ-induced neurotoxicity is one of the therapeutic targets for AD.

Many naturally derived antioxidants have been found to be able to induce decline in Aβ-induced neurotoxicity, such as Ginsenoside Rg1 [7], curcumin [8], and ginkgo biloba [9]. Tanshinone IIA (Tan IIA) is one of major lipophilic component extracted from a well-known traditional herb *Radix salvia miltiorrhiza* (Danshen) [10]. Tan IIA has been reported to suppress the development of many diseases including cancer [11], cardiovascular diseases [12] and cerebrovascular diseases [13]. Tan IIA is able to exert neuroprotective effect via its anti-oxidant and anti-inflammatory actions. For example, Lin et al. [14] revealed that Tan IIA attenuated hydrogen peroxide-induced damage to the human umbilical vein endothelial cells through its anti-oxidant effect and CD40 anti-inflammatory approach. Tan IIA was confirmed to reduce Aβ-induced neurotoxicity by its antioxidative potential in cortical neuron [15]. Tan IIA decreased the risk of Aβ-induced neuroinflammation by inhibiting nitric oxide synthase (iNOS) and matrix metalloproteinase-2 (MMP-2) via the nuclear transcription factor kappa (NF-κB) pathway [16]. These previous studies have demonstrated Tan IIA possesses anti-oxidant and anti-inflammatory actions, as well as neuroprotective effects. However, the detailed neuroprotection mechanism of Tan IIA against Aβ-induced neurotoxicity has not been well elucidated. In the present study, the neuroprotective effects of Tan IIA against Aβ-induced neurotoxicity in SH-SY5Y cells were investigated. Furthermore, the underlying mechanism by which Tan IIA exerts its neuroprotective effects in SH-SY5Y cells was elaborated.

## Methods

### Cell culture

The human neuroblastoma SH-SY5Y cells obtained from the American Type Culture Collection (ATCC, Rockville, Maryland, USA) were grown in DMEM (Invitrogen, Carlsbad, CA, USA) supplemented with 10% fetal bovine serum (FBS, Invitrogen) and 1% penicillin/streptomycin (Invitrogen). Cells were maintained at 37 °C in humidified 5% CO_2_.

### Cell treatments

Tan IIA used in the study was purchased from Sigma Chemical Company (Sigma, St. Louis, Missouri, USA). Tan IIA was dissolved in dimethyl sulfoxide (DMSO) before use. Aβ (1–42) peptide monomer (rat/mouse) was purchased from Sigma (Sigma). Aβ Peptide was dissolved in 1% NH_4_OH/Water and stored in aliquots in tightly sealed vials at − 20 °C. The solution was equilibrated to room temperature for at least 1 h before use. To investigate the neurotoxicity of Aβ, SH-SY5Y cells were exposed to Aβ at various concentrations (0, 5, 10, 25, 50 μM) for 48 h. To detect the neuroprotective effects of Tan IIA against Aβ-induced toxicity, SH-SY5Y cells were pretreated with Tan IIA (5 or 10 μM) for 1 h, followed by exposure to Aβ (25 μM) for 48 h.

### Quantitative real-time PCR (qRT-PCR)

Total RNA was extracted from SH-SY5Y cells using Trizol (Invitrogen). ImProm-II reverse transcription system (Promega, Madison, WI, USA) was applied to generate the first strand cDNA. qRT-PCR was performed using SYBR Premix Ex Taq™ (Takara Bio, Shiga, Japan) on the 7500 Real Time PCR System (Applied Biosystems, Foster City, CA, USA). β-actin was used as endogenous controls. The relative level of mRNA was analyzed using the 2^−ΔΔCt^ method. The sequences used for the real-time PCR were as follows: IL-1β, sense: 5′-GCAATGAGGATGACTTGTTCTTTG-3′ and antisense: 5′-CAGAGGTCCAGGTCCTGGAA-3′; TNF-α, sense: 5′-ACCTCTCTCTAATCAGCCCTCT-3′ and antisense: 5′-GGGTTTGCTACAACATGGGCTA-3′; IL-6, sense: 5′-AGCCACTCACCTCTTCAGAAC-3′ and anti-sense: 5′-ACATGTCTCCTTTCTCAGGGC-3′ COX-2, sense: 5′-CCAGCACTTCACGCATCAGT-3′ and anti-sense: 5′-ACGCTGTCTAGCCAGAGTTTCAC-3′; mPGES-1, sense: 5′-CCAAGTGAGGCTGCGGAAGAA-3′ and anti-sense: 5′-GCTTCCCAGAGGATCTGCAGA-3′ β-actin, 5′-CCTGACTGACTACCTCATGAAG-3′ and anti-sense: 5′-GACGTAGCACAGCTT’CTCCTTA-3′.

### Western blot analysis

Western blot analysis was performed following previous protocols [17]. Briefly, proteins were separated on SDS-PAGE, electrotransferred to a nitrocellulose membrane, and detected by incubating with specific primary antibodies. The immunoreactive bands were visualized using the ECL detection kit (ECL, Pharmacia Biotech, Piscataway, NJ, USA). The anti-COX-2, anti-MCL-1, anti-Cyclin D1, and anti-β-actin antibodies were purchased from Santa Cruz Biotechnology (SantaCruz, CA, USA). β-actin was used as an endogenous control.

### Cell viability assay

SH-SY5Y cells were seeded into the 96-well plate at a density of 3 × 10^3^/well. Cell viability was analyzed using MTT assay (Sigma) after treatments described as above. The absorbance at 450 nm was then determined using a microtiter plate reader (Molecular Devices, Sunnyvale, CA, USA).

### ELISA assay

SH-SY5Y cells inoculated in 96-well plate were treated as stated above. Then, the culture supernatant was collected to measure the concentrations of TNF-α, IL-1β, IL-6 and PGE2 using Enzyme-Linked Immunosorbent Assay (ELISA) kits (Elisa biotech, Shanghai, China), according to the manufacturer’s instructions. Proteins were extracted from treated SH-SY5Y cells to detect the protein levels of caspase 3 using ELISA kits (Elisa biotech). Nuclear cell lysates were fractionated using the Active Motif Nuclear Extract Kit (Active Motif, Carlsbad, CA), according to the manufacturer’s instructions. Nuclear lysates (20 μg) were then assayed for NF-κB that bound to immobilized double-stranded DNA oligonucleotides containing NF-κB consensus sequences, using the 96-well TransAM NF-κB ELISA kit (Elisa biotech) according to the manufacturer’s instructions.

### Apoptosis assay

Apoptosis was assessed using an Annexin-V-FITC apoptosis detection kit (BD Biosciences, San Jose, CA, USA). Briefly, SH-SY5Y cells were seeded in 6-well plates. After indicated treatments as mentioned above, cells were harvested and stained with FITC-Annexin V and Propidium iodide (PI). Then, cells were subjected to the FACScan flow cytometer (Becton–Dickinson, Mountain View, CA, USA) with Cell Quest 3.2 (Becton–Dickinson).

### Statistical analysis

All data were presented as mean ± SD from three independent experiments. Statistical significance was determined by Student’s *t*-test or one-way ANOVA using SPSS 19.0 software (SPSS, Chicago, IL, USA). A *p* value < 0.05 was considered significant.

## Results

### Aβ treatment reduces cell viability and induces apoptosis in SH-SY5Y cells

**Fig. 1:**
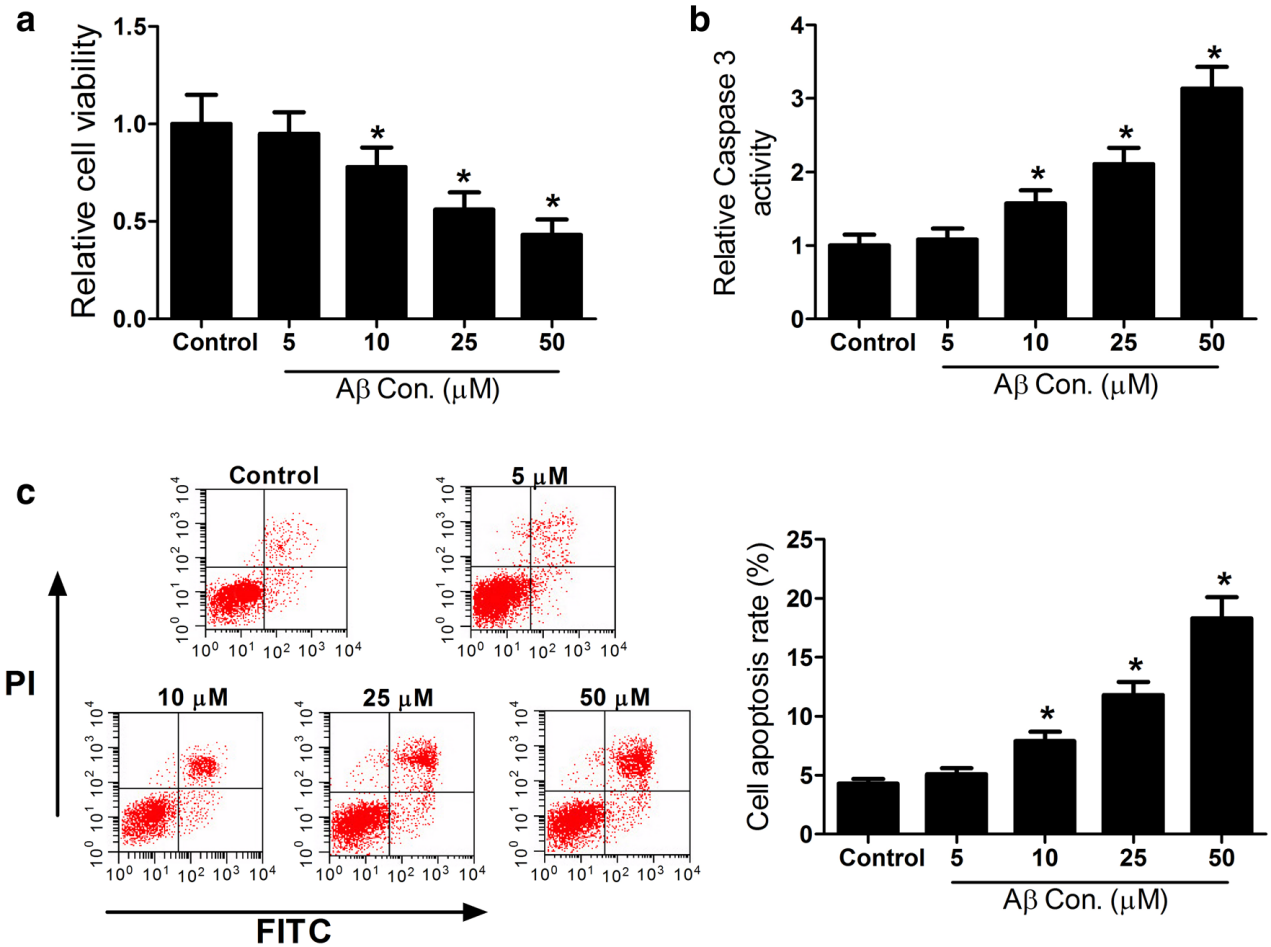
Aβ treatment suppressed cell viability and induced apoptosis in SH-SY5Y cells. SH-SY5Y cells were exposed to different concentrations (0, 5, 10, 25, 50 μM) of Aβ for 48 h. **a** MTT assay was conducted to determine the viability. **b** ELISA assay was performed to detect the caspase3 protein level. **c** Flow cytometry analysis was applied to determine apoptotic rate. * p < 0.05 vs. controls

To determine the neurotoxicity of Amyloid β-protein (Aβ), SH-SY5Y cells were exposed to different concentrations of Aβ (0, 5, 10, 25, 50 μM) for 48 h. MTT assay results indicated that Aβ resulted in a decrease in the viability of SH-SY5Y cells in a dose-dependent manner (Fig. 1a). It is well known that Aβ triggered neurotoxicity mainly by inducing neuronal apoptosis [18]. Moreover, activation of caspase-3 has been clarified to be a key event in neuronal apoptosis in response to Aβ exposure in vitro and in vivo [19, 20]. Thus, ELISA assay and flow cytometry assay was conducted to determine the caspase-3 protein level and cell apoptosis rates in SH-SY5Y cells treated with different concentrations of Aβ, respectively. As expected, Aβ treatment led to the increase in the caspase-3 protein level and apoptosis in SH-SY5Y cells in dose-dependent manners (Fig. 1b, c). All these results suggested that Aβ induced neurotoxicity by decreasing cell viability and inducing apoptosis in SH-SY5Y cells.

### Aβ treatment promotes expression of inflammatory factors in SH-SY5Y cells

**Fig. 2:**
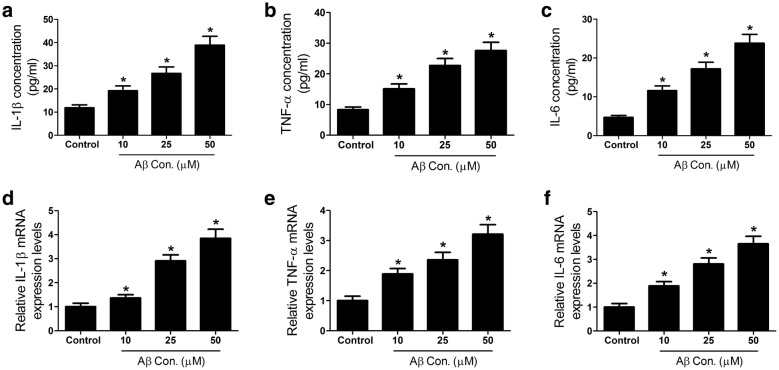
Aβ treatment increased the levels of inflammatory factors in SH-SY5Y cells. SH-SY5Y cells were treated with different concentrations (0, 10, 25, 50 μM) of Aβ for 48 h. **a**–**c** ELISA assay was carried out to measure the concentration of IL-1β, TNF-αand IL-6 in the culture supernatant. **d**–**f** qRT-PCR analysis was conducted to detect the mRNA expression of IL-1β, TNF-α and IL-6 in SH-SY5Y cells. *p < 0.05 vs. controls

Chronic neuroinflammation has been implied to be associated with many neurodegenerations including AD [21]. Therefore, we further explore the role of Aβ in neuroinflammation by treating SH-SY5Y cells with different concentrations of Aβ (0, 10, 25, 50 μM). ELISA assay was carried out to detect the levels of inflammatory factors (IL-1β, TNF-α, and IL-6) in the culture supernatant of SH-SY5Y cells. As presented in Fig. 2a–c, the supernatant of SH-SY5Y cells treated with Aβ displayed higher concentrations of IL-1β, TNF-α and IL-6 than the control groups in dose-dependent manners. Also, qRT-PCR analysis was performed to measure the mRNA expression of inflammatory factors. Consistently, expressions of IL-1β, TNF-α and IL-6 mRNA were elevated in dose-dependent manners in SH-SY5Y cells after Aβ treatment. Collectively, Aβ induced neuroinflammation by increasing the levels of inflammatory factors in SH-SY5Y cells.

### Aβ treatment leads to increase in expression of COX-2 and PGE2, as well as activation of NF-κB pathway in SH-SY5Y cells

**Fig. 3:**
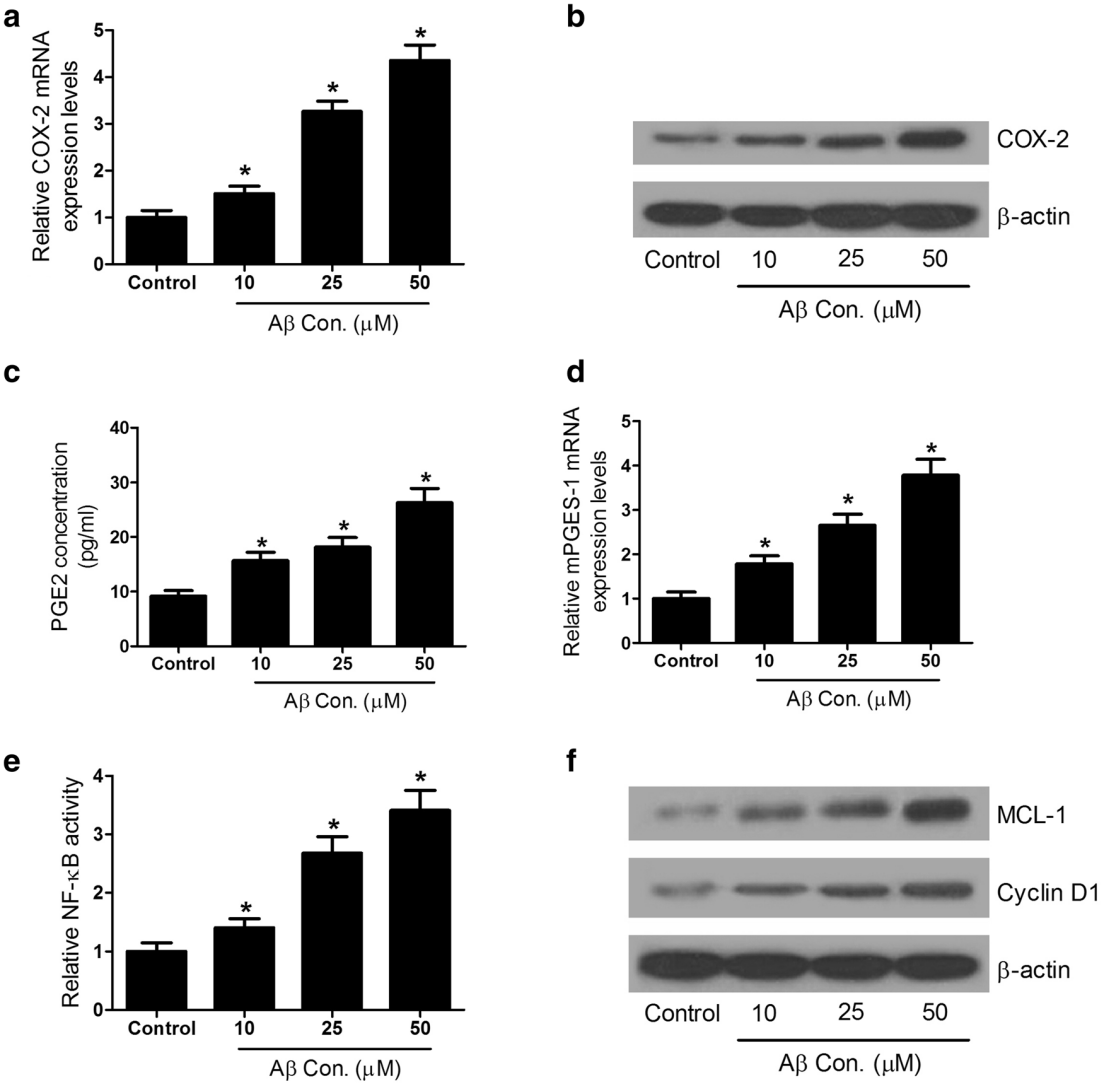
Aβ treatment promoted COX-2 expression and PGE2 secretion, and activated NF-κB pathway in SH-SY5Y cells. SH-SY5Y cells was exposed to different concentrations (0, 10, 25, 50 μM) of Aβ for 48 h. **a** qRT-PCR analysis of COX-2 mRNA expression. **b** Western blot analysis of COX-2 protein level. **c** ELISA assay of PGE2 concentration in culture supernatant. **d** qRT-PCR analysis of mPGES-1 mRNA expression. **e** ELISA assay of NF-κB protein levels. **f** Western blot analysis of MCL-1 and cyclin D1 protein levels. *p < 0.05 vs. controls

Previous studies confirmed that Prostaglandin E2 (PGE2) played pivotal functions in inflammation, which was catalyzed by a rate-limiting enzyme cyclooxygenase-2 (COX-2) [22, 23]. Therefore, the COX-2 expression and PGE2 secretion were determined by qRT-PCR, western blots and ELISA assay in SH-SY5Y cells treated with 0, 10, 25 or 50 μM Aβ. As shown in Fig. 3a, b, the mRNA and protein levels of COX-2 were significantly promoted by Aβ in SH-SY5Y cells in a dose-dependent manner. Moreover, PGE2 secretion and microsomal prostaglandin E synthase-1 (mPGES-1) mRNA expression were prominently increased in Aβ-treated SH-SY5Y cells (Fig. 3c, d).

A previous study reported that Aβ could activate the NF-κB pathway [24]. Thus, we further investigated the NF-κB levels in SH-SY5Y cells exposed to Aβ by ELISA assay and western blot analysis. As expected, Aβ treatment significantly increased the protein levels of NF-κB (Fig. 3e) and increased the expressions of MCL-1 and cyclin D1 (Fig. 3f), the downstream molecules of NF-κB pathway. Taken together, Aβ induced neurotoxicity by elevating COX-2 expression and PGE2 synthesis, and activating NF-κB pathway in SH-SY5Y cells.

### Tan IIA attenuates the effect of Aβ on cell viability and apoptosis in SH-SY5Y cells

**Fig. 4:**
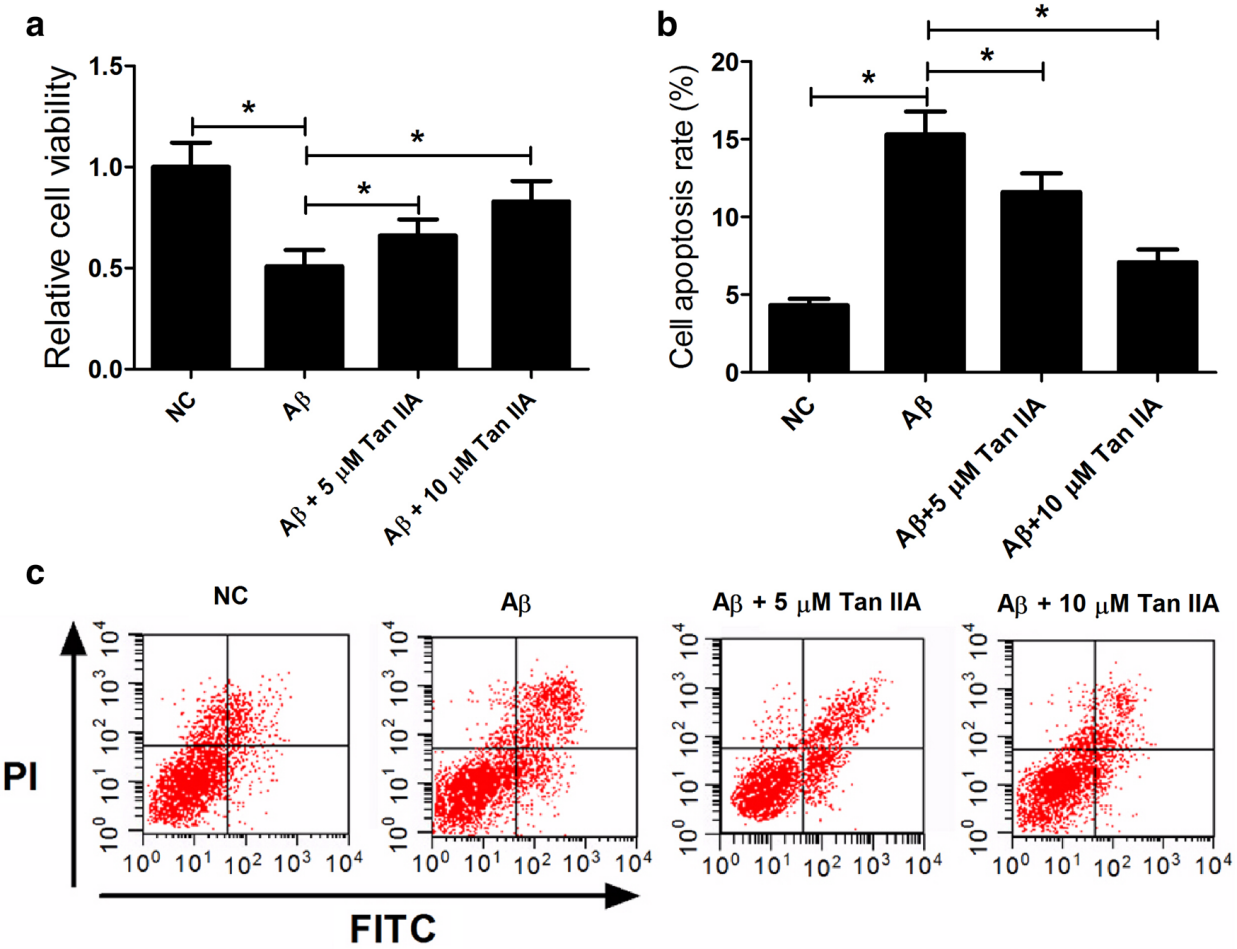
Tan IIA relieved the effect of Aβ on cell viability and apoptosis in SH-SY5Y cells. SH-SY5Y cells were pretreated with Tan IIA (5 or 10 μM) for 1 h prior to exposure to Aβ (25 μM) for 48 h. **a** MTT assay was performed to determine cell viability. **b**, **c** Flow cytometry analysis was conducted to assess apoptotic rates. *p < 0.05, **p < 0.01 vs. respective control

To investigate whether Tan IIA could prevent Aβ-induced neurotoxicity, SH-SY5Y cells were pretreated with Tan IIA (5 or 10 μM) for 1 h prior to exposure to Aβ (25 μM) for 48 h. Subsequently, MTT and flow cytometry analysis were conducted to determine the cell viability and apoptosis rate in SH-SY5Y cells. As shown in Fig. 4a, Aβ significantly decreased the cell viability, however, pretreatment with Tan IIA ameliorated Aβ-mediated suppression on cell viability. Moreover, pretreatment with Tan IIA clearly inhibited Aβ-induced apoptosis in SH-SY5Y cells (Fig. 4b, c). These results demonstrated that Tan IIA alleviated the neurotoxicity in SH-SY5Y cells through suppressing Aβ-mediated cell viability reduction and apoptosis induction.

### Tan IIA abates the inductive effect of Aβ on the inflammatory factors in SH-SY5Y cell

**Fig. 5:**
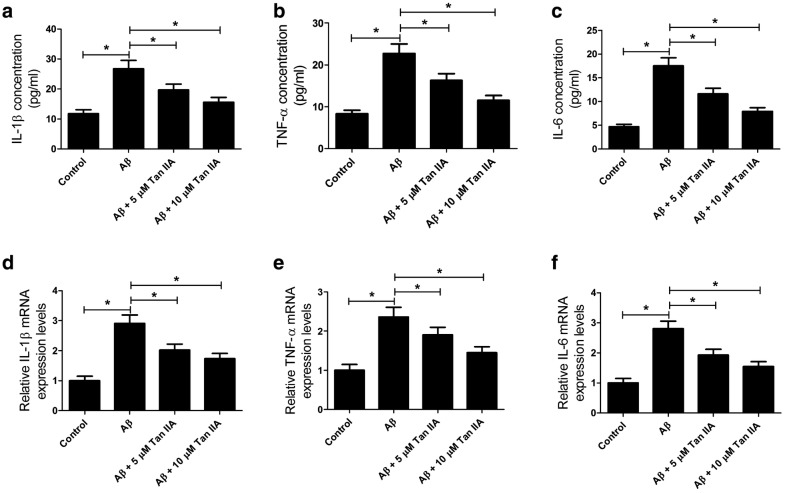
Tan IIA alleviated the inductive effect of Aβ on the expression of inflammatory factors in SH-SY5Y cells. SH-SY5Y cells were preconditioned with Tan IIA (5 or 10 μM) for 1 h followed by treatment with Aβ (25 μM) for 48 h. **a**–**c** ELISA assay examined the concentration of TNF-α, IL-1β and IL-6 in the culture supernatant. **d**–**f** qRT-PCR analysis evaluated the mRNA levels of TNF-α, IL-1β and IL-6 in SH-SY5Y cells. *p < 0.05, **p < 0.01 vs. respective control

To further confirm the mechanism of Tan IIA in Aβ-mediated neuroinflammation, SH-SY5Y cells were preconditioned with Tan IIA (5 or 10 μM) for 1 h followed by treatment with Aβ (25 μM) for 48 h. The concentration and mRNA levels of inflammatory factors including IL-1β, TNF-α and IL-6 were detected by ELISA assay and qRT-PCR analysis, respectively. As expected, pretreatment of SH-SY5Y cells with Tan IIA substantially prevented Aβ-induced increase in the concentrations of IL-1β, TNF-α and IL-6 in culture supernatant (Fig. 5a–c). Similarly, Aβ boosted the mRNA expressions of IL-1β, TNF-α and IL-6, which was greatly attenuated by Tan IIA pretreatment (Fig. 5d–f). All these results revealed that Tan IIA relieved Aβ-mediated neuroinflammation in SH-SY5Y cells.

### Tan IIA weakens Aβ-induced increase in COX-2 expression and PGE2 secretion, as well as activation of NF-κB pathway in SH-SY5Y cells

**Fig. 6:**
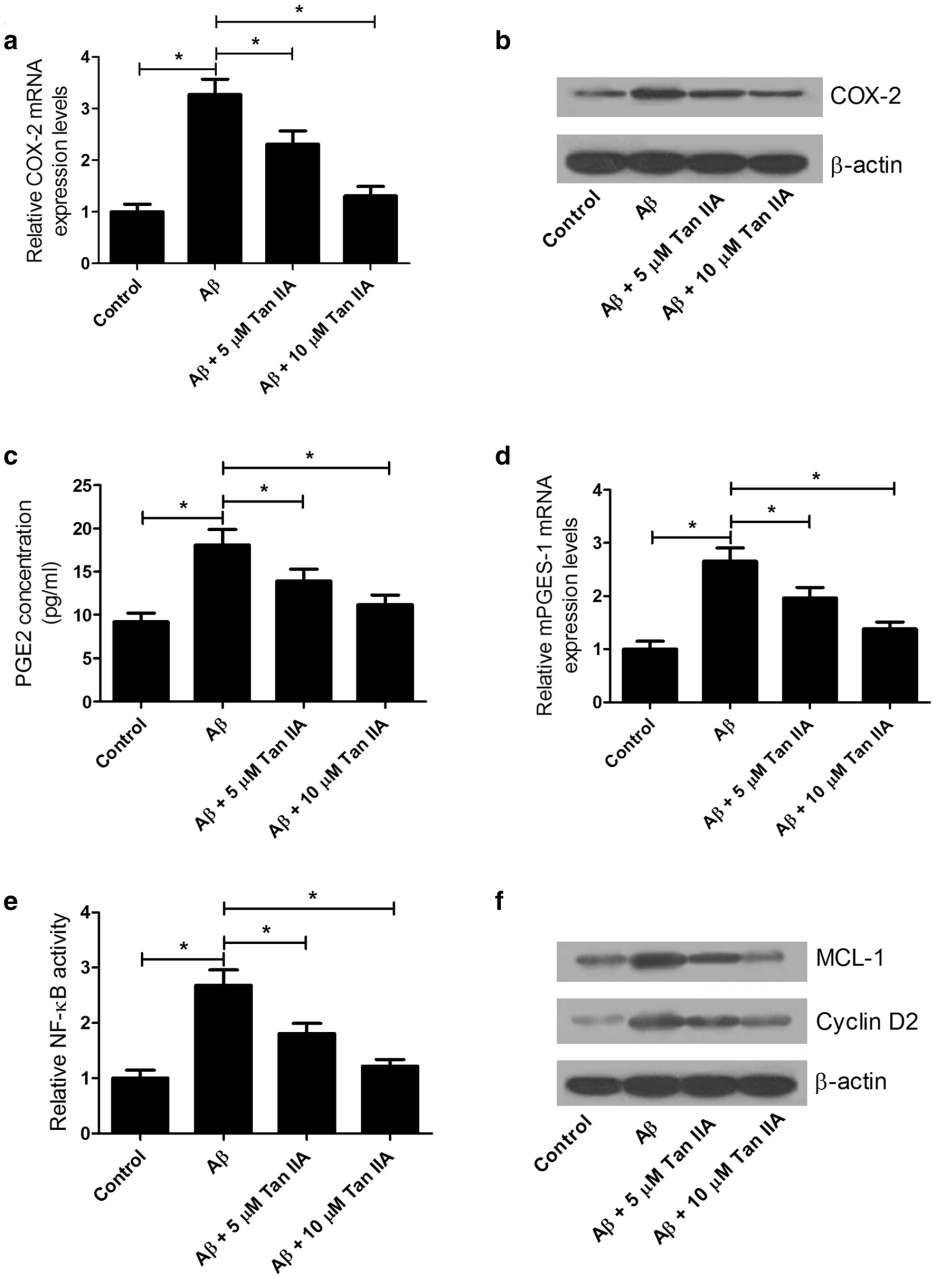
Tan IIA declined Aβ-induced increase in COX-2 expression and PGE2 secretion, and activation of NF-κB pathway in SH-SY5Y cells. SH-SY5Y cells were treated with Tan IIA (5 or 10 μM) for 1 h, then exposed to Aβ (25 μM) for 48 h. **a** The mRNA level of COX-2 was detected by qRT-PCR analysis. **b** The protein level of COX-2 was measured by western blot analysis. **c** The concentration of PGE2 in culture supernatant was determined by ELISA assay. **d** qRT-PCR analysis examined the mPGES-1 mRNA level. **e** NF-κB levels was determined by ELISA assay. **f** The protein levels of MCL-1 and cyclin D1 was detected by western blot analysis. *p < 0.05, **p < 0.01 vs. respective control

To further explore the mechanism by which Tan IIA ameliorated Aβ-induced neurotoxicity, COX-2 expression and PGE2 secretion in SH-SY5Y cells treated with either Aβ (25 μM) or in combined with Tan IIA (5 or 10 μM) were determined by qRT-PCR analysis, western blot analysis and ELISA assay. As displayed in Fig. 6a, b, Tan IIA pretreatment resulted in an apparent repression on COX-2 expression induced by Aβ at mRNA and protein level in SH-SY5Y cells. Moreover, Aβ treatment significantly increased PGE level in culture supernatant (Fig. 6c) and mPGES-1 mRNA expression in SH-SY5Y cells (Fig. 6d), while Tan IIA pretreatment depressed this effect. Also, Tan IIA pretreatment weakened Aβ-mediated increase of NF-κB, MCL-1 and cyclin D1 levels (Fig. 6e, f). All these data confirmed that Tan IIA attenuated Aβ-induced neurotoxicity by preventing COX-2 expression, PGE2 secretion, and NF-κB levels in SH-SY5Y cells.

## Discussion

In the present study, we investigated the neuroprotective effects of Tan IIA against Aβ-induced neurotoxicity in SH-SY5Y cells. Our study found that pretreatment with Tan IIA prevented Aβ-mediated reduction in cell viability, suppressed Aβ-triggered apoptosis and attenuated Aβ-induced release and expression of pro-inflammatory cytokines. Mechanistically, Tan IIA alleviated Aβ-induced COX-2 expression, PGE2 synthesis, and activation of NF-κB pathway. These results demonstrate that the neuroprotective effects of Tan IIA are most likely mediated by the NF-κB pathway.

Aβ is the major protein component of senile plaques, and the excess deposition of Aβ in the brain plays a central role in the etiology of AD. Therefore, to determine the mechanism by which Aβ induces neurotoxicity is important to identify potential molecular therapeutic targets for AD. Previous research efforts have reported that cultured neurons exhibited the characteristics of apoptosis when exposed to Aβ [15, 18]. The present study also demonstrated that Aβ treatment induced the apoptosis of SH-SY5Y cells in a dose-dependent manner. Moreover, neuroinflammation has been postulated to play a critical role in the development of AD pathology. Our study found that SH-SY5Y cells exposed to Aβ displayed higher concentration and expression of IL-1β, TNF-α and IL-6 than that of the control groups. In agreement with our results, Jiang et al. [25] revealed that Aβ could induce release of pro-inflammatory cytokines IL-1β, IL-10, and TNF-α. Previous studies confirmed that PGE2 played pivotal functions in inflammation and was elevated together with the expression of COX-2, a rate-limiting enzyme catalyzing PGE2 production, in the brain of AD patients [22, 23]. Consistently, our study found that COX-2 and PGE2 was significantly upregulated in Aβ-induced SH-SY5Y cells. Selective inhibition of COX-2 by NS398 acutely prevented the Aβ-induced impairment of synaptic transmission [26]. Suppression of COX-2 expression and PGE2 production decreased the neurotoxicity induced by TDP-43-deficient microglia through the MAPK/ERK pathway [27]. Additionally, Aβ was reported to activate the NF-κB pathway by selectively inducing the nuclear translocation of p65 and p50 subunits, and to promote apoptosis-related gene expressions, leading to nerve cell death [24]. Our study also confirmed that Aβ induced the activation of NF-κB pathway by improving NF-κB levels and upregulating MCL-1 and cyclin D1 expression. Collectively, our study demonstrated that Aβ induced neurotoxicity by increasing COX-2 expression and PGE2 synthesis, and activating NF-κB pathway in SH-SY5Y cells.

Tan IIA has been reported to be effective in many diseases due to its anti-oxidant and anti-inflammation effects. Qian et al. [18] revealed that the neuroprotective effect of Tan IIA against Aβ-induced cytotoxicity was mediated by activation of the Bcl-xL pathway. Parallelly, another study demonstrated that Tan IIA attenuated Aβ-induced neurotoxicity through calpain and the p35/Cdk5 pathway [28]. Tan IIA has been indicated to exert neuroprotective effects by promoting RACK1 and repressing autophagy in the hippocampus of mice [29]. A recent paper showed that Tan IIA inhibited the viability of glioma cells and induce apoptosis and autophagy possibly via inactivation of the PI3K/Akt/mTOR signal pathway [30]. In the present study, we found that Tan IIA ameliorated Aβ-induced neurotoxicity in the SH-SY5Y cells by preventing Aβ-mediated cell viability reduction, apoptosis induction and pro-inflammatory effect. Mechanically, Tan IIA exerted its neuroprotective action by suppressing Aβ-induced increase of COX-2 expression and PGE2 synthesis, and activation of NF-κB pathway.

Increasing evidence suggests that NF-κB pathway contributed to the up-regulation of pro-inflammatory and cytotoxic genes during the degenerative process of disease [31, 32], indicating its critical role in nerve injuries. Tan IIA exerts anti-inflammatory and immune-regulating effects on human umbilical vein endothelial cells and vulnerable atherosclerotic plaque partially via suppressing NF-κB signal pathway [33, 34]. Moreover, Sodium Tanshinone IIA sulfonate attenuates hemorrhagic shock-induced organ damages by NF-κB pathway [35]. Tan IIA could inhibit angiogenesis through down regulation of COX-2 in human colorectal cancer [36]. Our study found elevated COX-2 expression and PGE2 synthesis and activated NF-κB pathway in SH-SY5Y cells exposed to Aβ. Moreover, the regulatory relationship between COX-2, PGE2 and NF-κB pathway is not well known. COX-2 and PGE2 synthase have been shown to be strongly activated during neuroinflammation [37]. Additionally, the promoter region of the COX-2 gene contained the binding site of NF-κB, which was the main transcription factor involved in COX-2 gene expression during inflammatory processes [38, 39]. NF-κB activation contributed to the induction of COX-2 expression and PGE2 production in macrophages [40]. All these reports combined with our results prompted us to conclude that Tan IIA alleviated Aβ-induced neurotoxicity by down-regulating COX-2-PGE2 expression via inactivation of NF-κB pathway in SH-SY5Y cells.

## Conclusion

In summary, our study demonstrated that Tan IIA attenuated Aβ-induced neurotoxicity by down-regulating COX-2 expression and PGE2 synthesis, and inactivation of NF-κB pathway in SH-SY5Y cells. These findings suggest that Tan IIA protect SH-SY5Y cells against Aβ-induced neurotoxicity most likely via NF-κB pathway.

## Data Availability

Not applicable.
